# NLRC5 promotes endometrial carcinoma progression by regulating NF-κB pathway-mediated mismatch repair gene deficiency

**DOI:** 10.1038/s41598-024-63457-2

**Published:** 2024-05-30

**Authors:** Xiaojing Liu, Haiqing Zhu, Bao Guo, Jiahua Chen, Junhui Zhang, Tao Wang, Jing Zhang, Wenjun Shan, Junchi Zou, Yunxia Cao, Bing Wei, Lei Zhan

**Affiliations:** 1 Department of Obstetrics and Gynecology, The Second Affiliated Hospital of Anhui Medical University, No 678 Furong Road, Hefei, 230022 Anhui China; 2 Department of Obstetrics and Gynecology, The Frist Affiliated Hospital of Anhui Medical University, Hefei, Anhui China; 3 Department of Oncology, The Second Affiliated Hospital of Anhui Medical University, Hefei, Anhui China

**Keywords:** Endometrial carcinoma, NLR family CARD domain-containing 5, Mismatch repair gene deficiency, Nuclear factor-kappaB, Cancer, Cell biology

## Abstract

The innate immune molecule NLR family CARD domain-containing 5 (NLRC5) plays a significant role in endometrial carcinoma (EC) immunosurveillance. However, NLRC5 also plays a protumor role in EC cells. Mismatch repair gene deficiency (dMMR) can enable tumors to grow faster and also can exhibit high sensitivity to immune checkpoint inhibitors. In this study, we attempted to determine whether NLRC5-mediated protumor role in EC is via the regulation of dMMR. Our findings revealed that NLRC5 promoted the proliferation, migration, and invasion abilities of EC cells and induced the dMMR status of EC in vivo and in vitro. Furthermore, the mechanism underlying NLRC5 regulated dMMR was also verified. We first found NLRC5 could suppress nuclear factor-kappaB (NF-κB) pathway in EC cells. Then we validated that the positive effect of NLRC5 in dMMR was restricted when NF-κB was activated by lipopolysaccharides in NLRC5-overexpression EC cell lines. In conclusion, our present study confirmed the novel NLRC5/NF-κB/MMR regulatory mechanism of the protumor effect of NLRC5 on EC cells, thereby suggesting that the NLRC5-mediated protumor in EC was depend on the function of MMR.

## Introduction

Endometrial carcinoma (EC), which is the sixth most common cancer among women worldwide, usually occurs in elderly women. In recent years, the treatment of EC has received increasing attention owing to the increase in the incidence of EC and the younger age of disease onset. Surgery, radiotherapy, and chemotherapy, the main treatments for EC, are not effective in the case of high-grade and recurrent disease^1^. Therefore, studying the pathogenesis of EC and identifying potential therapeutic targets is of immense social significance and medical value.

According to mutation load and somatic copy number variation, The Cancer Genome Atlas (TCGA) Research Network categorized EC into four different molecular subgroups: namely, polymerase-epsilon (POLE) ultra-mutated, mismatch repair gene deficiency (dMMR) or microsatellite instability hypermutated (MSI-H), a copy-number low group with a low mutational burden, and a copy-number high serous-like group^2,3^. The dMMR tumor accounts for 17–33% of all ECs^4^. The DNA mismatch repair (MMR) gene is composed of MLH1, MSH2, MSH6, and PMS2, which can maintain high fidelity and genomic stability of DNA replication and reduce spontaneous mutation. Any mutation or loss in these four genes is called dMMR, and manifests as genome instability (microsatellite instability, MSI). This instability either activates or inactivates mutations in various cancer-related genes that affect cell growth or survival, which accelerates the occurrence and development of tumors^5^. A large number of mutations in dMMR tumors, which leads to increased tumor mutation burden (TMB), results in the production of related new antigens. Therefore, dMMR tumors often show substantial morphological heterogeneity^6^, with high infiltration of CD8^+^ T cells^7^, and high sensitivity to immune checkpoint inhibitors^8,9^. Therefore, one the one hand, dMMR is one of the most important pathogenesis for tumor development. One the other hand, dMMR seems contribute to oncotherapy by improving the sensitivity of tumor immunotherapy. The dMMR is often associated with promoter methylation and inflammation^10^, study have also found that MMR may be regulated by inflammatory pathways induced by NF-κB activation in oral cancer^11^. However, the regulation of dMMR in EC is yet to be elucidated, and understanding the upstream events controlling MMR is particularly important for formulating comprehensive strategies and guiding clinical cancer treatment.

Nucleotide-binding oligomerization (NOD)-like receptor (NLR) family CARD domain 5 (NLRC5) has been confirmed to be an important regulator of innate and adaptive immunity^12,13^, which is involved in immune and inflammatory responses. NLRC5 overexpression is associated with CD8^+^ cytotoxic T cell activity^14,15^, promote the immunogenicity of tumors in human cancers^16^, studies showed that in EC, NLRC5 could inhibit tumor progression by activating immunosurveillance^14,17^. Paradoxically, previous studies have shown that NLRC5 promotes tumor cell proliferation, migration, and invasion in various cancers, including renal clear cell carcinoma, hepatocellular carcinoma, and gastric cancer^18–21^. Moreover, our previous study has suggested that NLRC5 promotes the progression of tumor by inducing the expression of programmed death-ligand 1 (PD-L1) in EC cells^22^. Therefore, the overlapping role of NLRC5 in EC remains undetermined.

In some diseases such as atherosclerosis, osteoarthritis, and acute lung injury, overexpression of NLRC5 can inhibit the activation of inflammation, which may be linked to the negative regulatory of NLRC5 on interferon (IFN)-I and nuclear factor-kappaB (NF-κB)^23–25^. NF-κB is a ubiquitous nuclear transcription factor, and its regulation disorder is related to several diseases. The NF-κB signal pathway plays a key role in promoting tumor pathogenesis, as well as lung, autoimmune, and cardiovascular diseases^26^. Previous studies have shown that NLRC5 can specifically bind to the IκB kinase (IKK) complex (IKK-α/IKK-β) and inhibit the expression of NF-κB^27,28^. In mice deficient in the NLRC5 gene, the deletion of NLRC5 significantly increased the phosphorylation of IKK in peritoneal macrophages and enhanced the activation of NF-κB induced by toll-like receptors and the production of various proinflammatory factors^29^. Our present study aim to validate whether NLRC5 plays the protumor role in EC cells is depend on dMMR. We also evaluated whether NLRC5 plays role in dMMR in EC cells is through regulating NF-κB pathway. These results may help to explain why the innate immune molecule NLRC5 could also play a protumor role in EC cells, and providing potential precise treatment for EC depend on NLRC5.

## Materials and methods

### Bioinformatics analysis

The UCEC dataset was downloaded from the Cancer Genome Atlas database (TCGA, https://portal.gdc.cancer.gov/), which included 554 cases of endometrial cancer tissue and 35 cases of adjacent tissue. The correlation of NLRC5 with MSH6 and MSH2 protein expression in the EC was analyzed using the GEPIA database (http://gepia.cancer-pku.cn). Differentially expressed genes (DEGs) associated with NLRC5 gene in EC were analyzed using the LinkedOmics database (http://www.linkedomics.org), and then the 55 associated DEGs with correlation coefficients (cor) > 0.5 were identified in the STRING database (https://cn.string-db.org/) to construct a protein interaction network and screened the 10 hub genes with the most interactions using CYTOSCAPE 3.9.1 software (https://cytoscape.org/). Finally, GO functional enrichment analysis and KEGG pathway enrichment analysis were performed for the 10 hub genes using R4.1.0 (https://www.r-project.org/) and the enrichment results were visualized (the r packages used were "clusterProfiler" and "ggplot2").

### Cell culture and treatment

The human endometrial adenocarcinoma cell line (HEC-1B) was acquired from the American Type Culture Collection (ATCC, HTB113), while the endometrioid adenocarcinoma cell line Ishikawa was purchased from the European Collection of Authenticated Cell Cultures (ECACC, 99040201). These cell lines were maintained in a 37 °C incubator with a 5% CO_2_ atmosphere in high‑glucose Dulbecco's modified Eagle's medium (DMEM; Basalmedia, China) supplemented with 10% heat‐inactivated fetal bovine serum (FBS; Wisent, China). NF-κB was activated by lipopolysaccharides (LPS, Sigma-Aldrich, America).

### Cell lentivirus transfection

NLRC5 overexpression lentivirus was purchased from Genepharma (Shanghai, China). Lentivirus was added in appropriate titers to the EC cell lines HEC-1B and Ishikawa. Polybrene was added at the final concentration of 5 μg/mL. After 4–5 days of transfection, puromycin was used to screen the cells expressing lentivirus. The overexpression efficiency of *NLRC5* was verified by Western blotting and qRT-PCR.

### Western blot

Proteins were extracted with Ripa lysate containing 1% PMSF and phosphatase inhibitors from cells. Proteins was quantified and then added to loading buffer and boiled. Add an equal amount of protein sample to the electrophoresis tank. When the target protein was NLRC5, the protein load of HEC-1B and Ishikawa cell line was 30 μg/well. We found that the expression of MMR in the Ishikawa cell line was much lower than that in HEC-1B cell line in our experiment, in order to get more obvious data, when the target protein was not NLRC5, the protein load of HEC-1B is 15 μg/well; and that of Ishikawa cell line was 30 μg /well. The protein samples were electrophoresed in sodium dodecyl sulfate (SDS)-polyacrylamide gel for 2 h, transferred to PVDF membranes and blocked with skim milk blocking solution for 1 h. The blots were cut according to the molecular weight, then react with the primary antibody and incubate overnight at 4 °C. Primary antibodies used in this study were as follows: NLRC5 (cat. no. DF13672, Affinity), MSH6 (cat. no. DF6165, Affinity), MSH2 (cat. no. DF6257, Affinity), PMS2 (cat. no. DF3627, Affinity), MLH1 (cat. no. DF6057, Affinity), NF-κB p65 (cat. no.AF5006, Affinity), phosphorylated NF-κB p65 (cat. no. AF2006, Affinity) and β-Actin (cat. no. GB15003, Servicebio). The next day, the primary antibody was washed with TBST, and then the corresponding secondary antibody (cat. no. S0001, Affinity) was used for reaction, and incubated at room temperature for 1 h. Finally, use a gel imager to develop and obtain protein band. Although we did not retain the original Western Blot images showing full length membranes, the images we provide all have corresponding brightfield images in the supplementary material, which cropped with marks, and have clear boundaries, which can also prove the authenticity and validity of our data. The relative expression of proteins was analyzed using ImageJ software (version 1.8.0; https://imagej.net/ij/).

### qRT-PCR

Total RNA was extracted from the cells with the Trizol reagent (Takara, Japan) according to the manufacturer's instructions. cDNA was obtained by reverse transcription using a mixture of RNA in the PCR instrument. According to the kit instructions, cDNA, primers, and TBgreen (Takara, Japan) were added to the 8-strip tube, and the reaction was conducted in the fluorescence quantitative PCR instrument AgilentMX3000P. The reaction system was composed of 10 μL, of the mix with three complex holes in each group. The relative expression of mRNA was calculated by the 2^−ΔΔCt^ method. The primers obtained from Sangon Biotech (China) were:

- NLRC5-F 5ʹ-AACGAGACCTTGGACCCTGAA-3ʹ,
- NLRC5-R 5ʹ-GCTGGTGAACCCATCATCATAG-3ʹ;
- MSH6-F 5ʹ-TCATCCGCGAGAAAGGGAAAT-3ʹ,
- MSH6-R 5ʹ-TCATCCGCGAGAAAGGGAAAT-3ʹ;
- MSH2-F 5ʹ-AGTCAGAGCCCTTAACCTTTTTC-3ʹ,
- MSH2-R 5ʹ-GAGAGGCTGCTTAATCCACTG-3ʹ;
- PMS2-F 5ʹ-CAATGGATGTGGGGTAGAAGAAG-3ʹ,
- PMS2-R 5ʹ-GTTAGGTCGGCAAACTCTTGAAT-3ʹ;
- MLH1-F 5ʹ-CAACAAGTCTGACCTCGTCTTC-3ʹ,
- MLH1-R 5ʹ-CCGGGAATCTGTACGAACCAT-3ʹ;
- β-actin-F 5ʹ-CACCCAGCACAATGAAGATCAAGAT-3ʹ,
- β-actin-R 5ʹ-CAGTTTTTAAATCCTGAGTCAAGC-3ʹ.

### Cell counting kit-8 (CCK-8) assay

Cell viability was measured using the CCK-8 kit (Biosharp Life Sciences, China). The treated cells were inoculated in a 96-well plate at the density of 2000 cells/well and then cultured in an incubator for 24, 48, and 72 h. Then, 10% CCK8 solution was added to each well and the plate was incubated for 40 min away from direct light. The absorbance at 450 nm was detected with a microplate reader (Thermo Fisher Scientific, Inc.). All experiments were conducted in triplicate.

### Scar-wound healing assay

The cells were planted in 6-well plates until they reached a 90–95% confluence. A cross-shaped wound was created with a sterile 200-μL pipette tip. The remaining cells were gently washed thrice with cold PBS. The relevant images were collected at 0 h and 24 h after marking to observe the extent of wound healing.

### Transwell assay

In the migration experiment, 4 × 10^4^ cells were resuspended in a serum-free medium and directly implanted into the upper chamber of the Transwell chamber. In the invasion experiment, 2 × 10^6^ cells were planted in the upper chamber and covered with 50 μL matrix-glue (BD, America), while 600 μL of the medium containing 20% FBS was installed in the lower chamber. After 36 h of incubation, the adherent cells were fixed, and the images were collected after crystal violet staining for 10 min.

### Xenograft experiment

A total of 7 female BALB/c nude mice were acquired from SPF (Beijing) Laboratory Animal Technology Co. Ltd. Mice were randomly divided into a control group (n = 3), and a NLRC5-OE group (n = 4). The treated HEC-1B cells and NLRC5-overexpressed HEC-1B cells were subcutaneously inoculated into the right axilla of the experimental mice (age: 5 weeks, weight: 19–23 g). The cervical dislocation was performed to sacrifice the mice after 21 days, and their tumor was removed, weighed and then photographed. The tumor volumes were calculated using the following formula: tumor volume (mm3) = (ab2)/2 [a: the longest axis (mm), b: the shortest axis (mm)]. The tumor tissues were stained with hematoxylin and eosin (HE) as well as by immunohistochemical methods. All animal feeding and in vivo experimental procedures were approved by the Regulations on Laboratory Animal Management of the Animal Experimental Department of Anhui Medical University (Ethical committee approval No: LLSC201800855). All the methods were performed in accordance with the relevant Ethical guidelines and regulations. The study was carried out in compliance with the ARRIVE guidelines.

### Immunohistochemistry assay and HE staining

For the immunohistochemistry assay, the tissues obtained from the xenograft models were deparaffinized and then rehydrated with xylene and alcohol. After 15 min of microwaving to accomplish antigen recovery, the endogenous peroxidase activity was blocked with 0.3% hydrogen peroxide, after which the slides were incubated overnight with antibodies against MSH2 (cat. no. DF6257, Affinity), PMS2 (cat. no. DF4351, Affinity), and MLH1 (cat. no. DF6057, Affinity) at 4 °C and then with HRP-conjugated secondary antibody at 37 °C for 30 min. Next, the signals were detected with DAB and the nuclei were counterstained with hematoxylin. All antibodies were diluted in the ratio of 1:200. Dark-brown staining indicated a positive reaction. The intensity of dark-brown staining was analyzed with the ImageJ software.

For HE staining, the tissues were stained in an aqueous hematoxylin solution for several minutes, separated in acid and ammonia, and then dehydrated in 70% and 90% alcohol for 10 min each. Finally, the tissues were stained with an alcohol-eosin staining solution for 2–3 min.

### Statistical methods

Data were analyzed by GraphpadPrism (version 9.0, https://www.graphpad.com/). Student’s *t*-test was used to compare the data between two independent samples, and one-way ANOVA was used to compare the data of three groups and more than three groups. The data was expressed as mean ± standard deviation (SD). *P* < 0.05 was considered statistically significant.

### Ethics approval and consent to participate

All animal feeding and in vivo experimental procedures were approved by the Regulations on Laboratory Animal Management of the Animal Experimental Department of Anhui Medical University (Ethical committee approval No: LLSC201800855). All the methods were performed in accordance with the relevant Ethical guidelines and regulations. The study was carried out in compliance with the ARRIVE guidelines.

## Results

### NLRC5 enhances the progression of EC in vivo and in vitro

**Figure 1:**
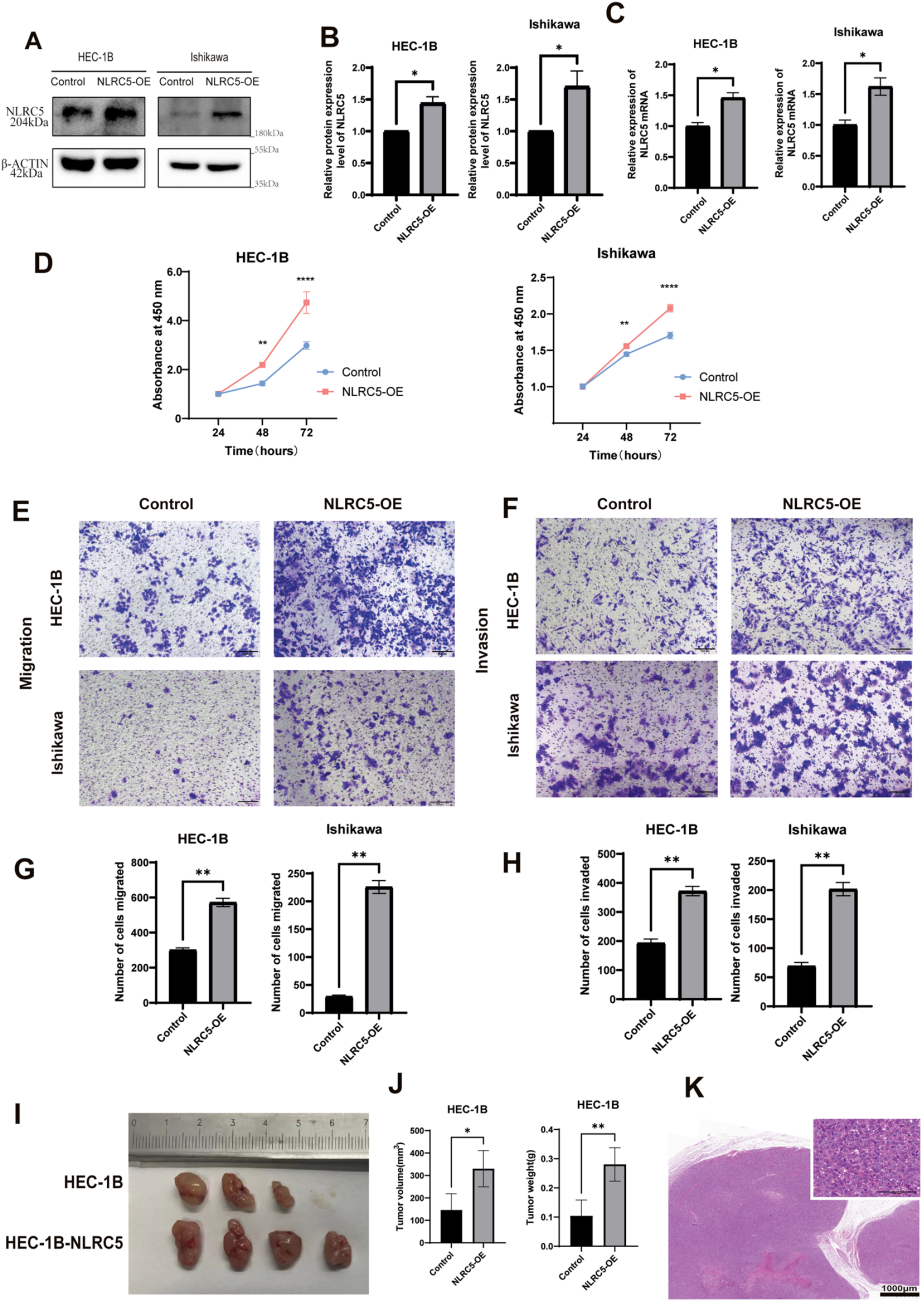
NLRC5 promotes the migration and invasion of EC cells and enhances the proliferation of EC in vivo and in vitro. (**A**,**B**) The proteins expression levels of NLRC5 in HEC-1B and Ishikawa cell lines were examined by western blot (mean ± SD, n = 3). (**C**) The mRNA expression levels of NLRC5 in HEC-1B and Ishikawa cell lines were examined by qRT-PCR (mean ± SD, n = 3). (**D**) The proliferation of HEC-1B cells and Ishikawa cells was measured via CCK-8 at 24,48 and 72 h (mean ± SD, n = 3). (**E**,**F**) Transwell assay detect the ability of cell migration (Scale bar = 100 μm, mean ± SD, n = 3). (**G**,**H**) Transwell assay detect the ability of cell invasion (Scale bar = 100 μm, mean ± SD, n = 3). (**I**) Representative tumor images. **J**) The average tumor volume and weight of nude mice. (**K**) Representative hematoxylin and eosin (HE) stained images of tumors (Scale bar: up 100 μm and down 1000 μm). The corresponding brightfield images are included in the supplementary material. Data represent mean ± SD, n = 3, *P < 0.05 versus control, **P < 0.01 versus control, ****P < 0.0001 versus control group.

First, NLRC5-overexpressing lentivirus was transfected into HEC-1B and Ishikawa cell lines, and the overexpression of NLRC5 was confirmed (Fig. 1A–C). Later, the CCK-8 kit was used to detect the differences in cell viability between the control and NLRC5-overexpression groups. The results showed that NLRC5 overexpression significantly promoted cell viability in both HEC-1B and Ishikawa cell lines (Fig. 1D). Next, transwell assays were used to compare the ability of migration and invasion between NLRC5-OE group and control group, results showed that NLRC5 promoted the migration and invasion abilities of EC cells (Fig. 1E–H). Finally, animal xenograft models of subcutaneous transplantation of HEC-1B cell lines were established. After 21 days of feeding, NLRC5 was observed to significantly promote the growth of tumors in vivo (Fig. 1I–K).

### Overexpression of NLRC5 induces dMMR of EC in vivo and in vitro.

**Figure 2:**
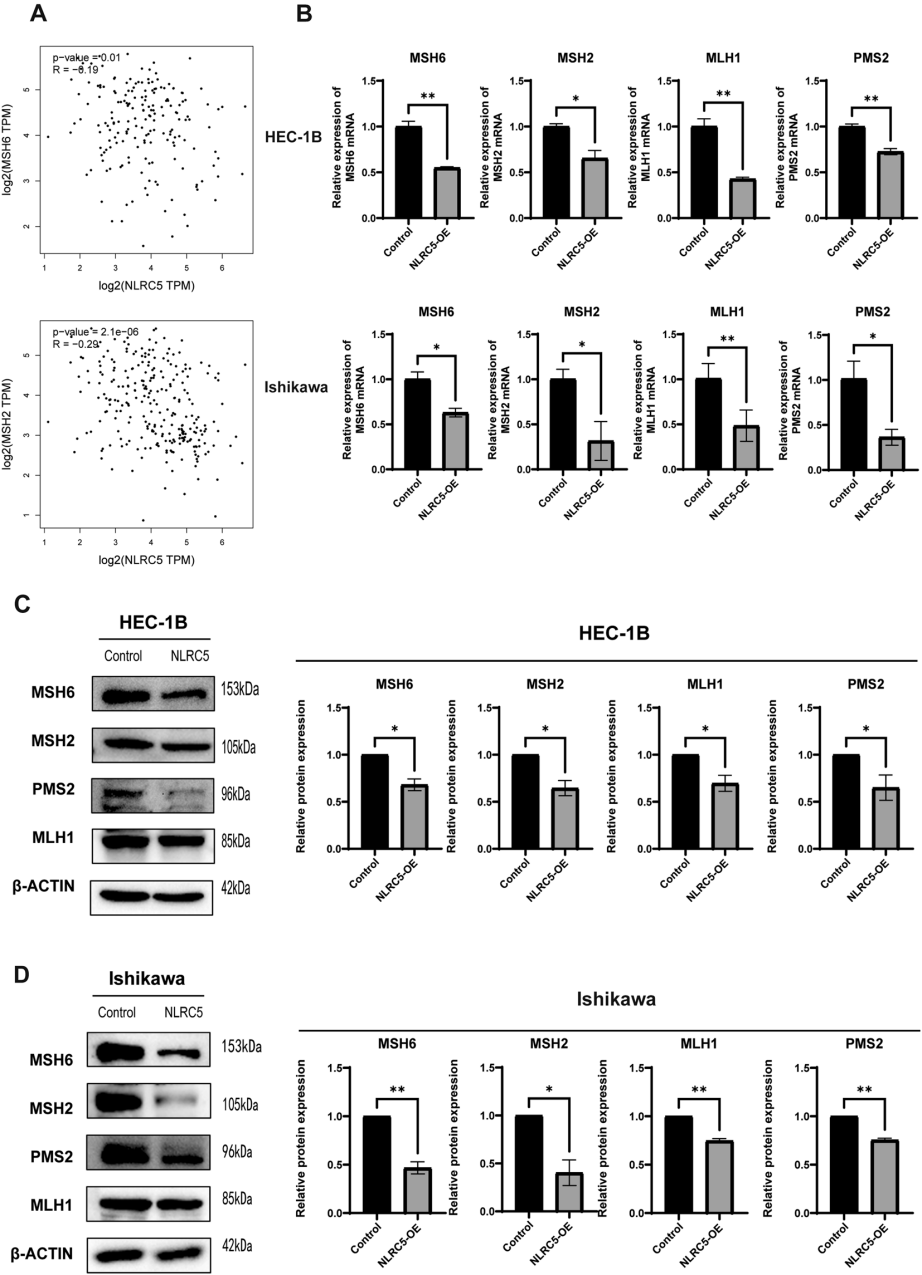
NLRC5 inhibits the expression of MMR genes in EC cells. (**A**) The correlation between NLRC5 and MSH6, MSH2 in EC was analyzed on GEPIA website. (**B**) The mRNA expression levels of NLRC5 in HEC-1B and Ishikawa cell lines were examined by qRT-PCR (mean ± SD, n = 3). (**C**) The protein expression levels of NLRC5 in HEC-1B and Ishikawa cell lines were examined by western blot (mean ± SD, n = 3). (**D**) The protein expression levels were quantified using the ImageJ software and normalized using β-actin protein levels (mean ± SD, n = 3). The corresponding brightfield images are included in the supplementary material. Data represent mean ± SD, *P < 0.05 versus control, **P < 0.01 versus control.

**Figure 3:**
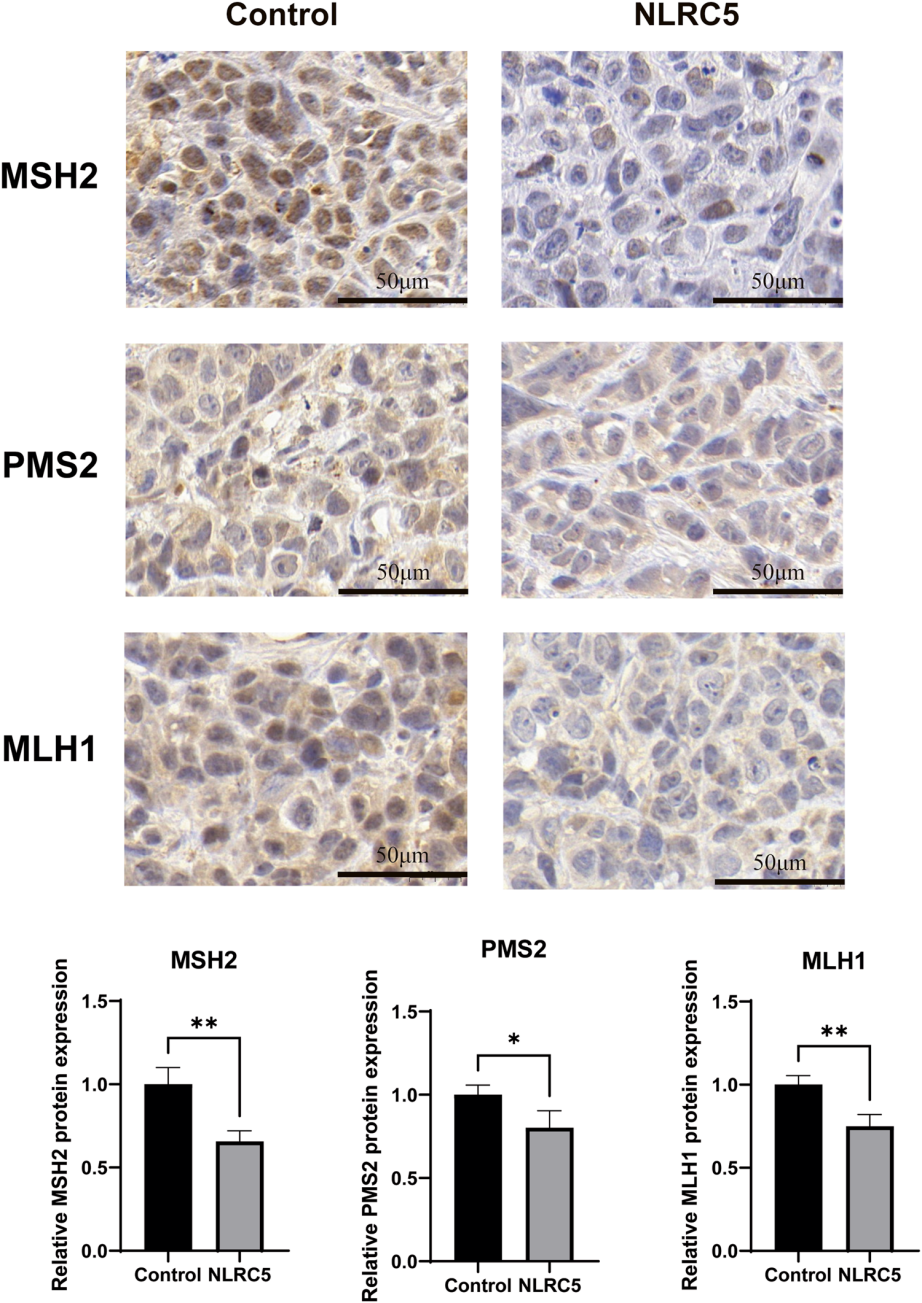
NLRC4 inhibits the expression of MSH2, PMS2, MLH1 genes in vivo. Representative images of results of immunohistochemical (IHC) detection of MSH2, PMS2, MLH1 gene expression of xenograft tumors. (Scale bar = 100 μm mean ± SD, n = 3). The protein expression levels were quantified using the Image J software. Data represent mean ± SD, *P < 0.05 versus control, **P < 0.01 versus control, ***P < 0.001 versus control.

Whether NLRC5 promoted cell proliferation, migration, and invasion was determined by inducing dMMR of EC. First, Gene Expression Profiling Interactive Analysis (GEPIA) database was used to analyze the correlation between NLRC5 and MMR genes in EC tissues. The results suggested that NLRC5 was weakly negatively correlated with MSH6 and MSH2 (Fig. 2A). To further verify whether NLRC5 can induce dMMR status of EC, qRT-PCR and western blot were used to detect the expressions of MMR genes, including MSH6, MSH2, MLH1, and PMS2. The findings revealed that the expressions of MSH6, MSH2, MLH1, and PMS2 were inhibited when NLRC5 was overexpressed (Fig. 2B–D), which indicated that NLRC5 overexpression induced dMMR status of EC cell lines. Subsequently, immunohistochemistry (IHC) of tumors stripped from mice were used to detect the MMR gene expression changes in vivo. Although we have not detected the valid data of MSH6 expression, the expressions of MSH2, PMS2 and MLH1 were significantly inhibited in vivo (Fig. 3). It attests that NLRC5 induces dMMR of EC both in vivo and in vitro.

### NLRC5 suppresses the NF-κB pathway in EC

**Figure 4:**
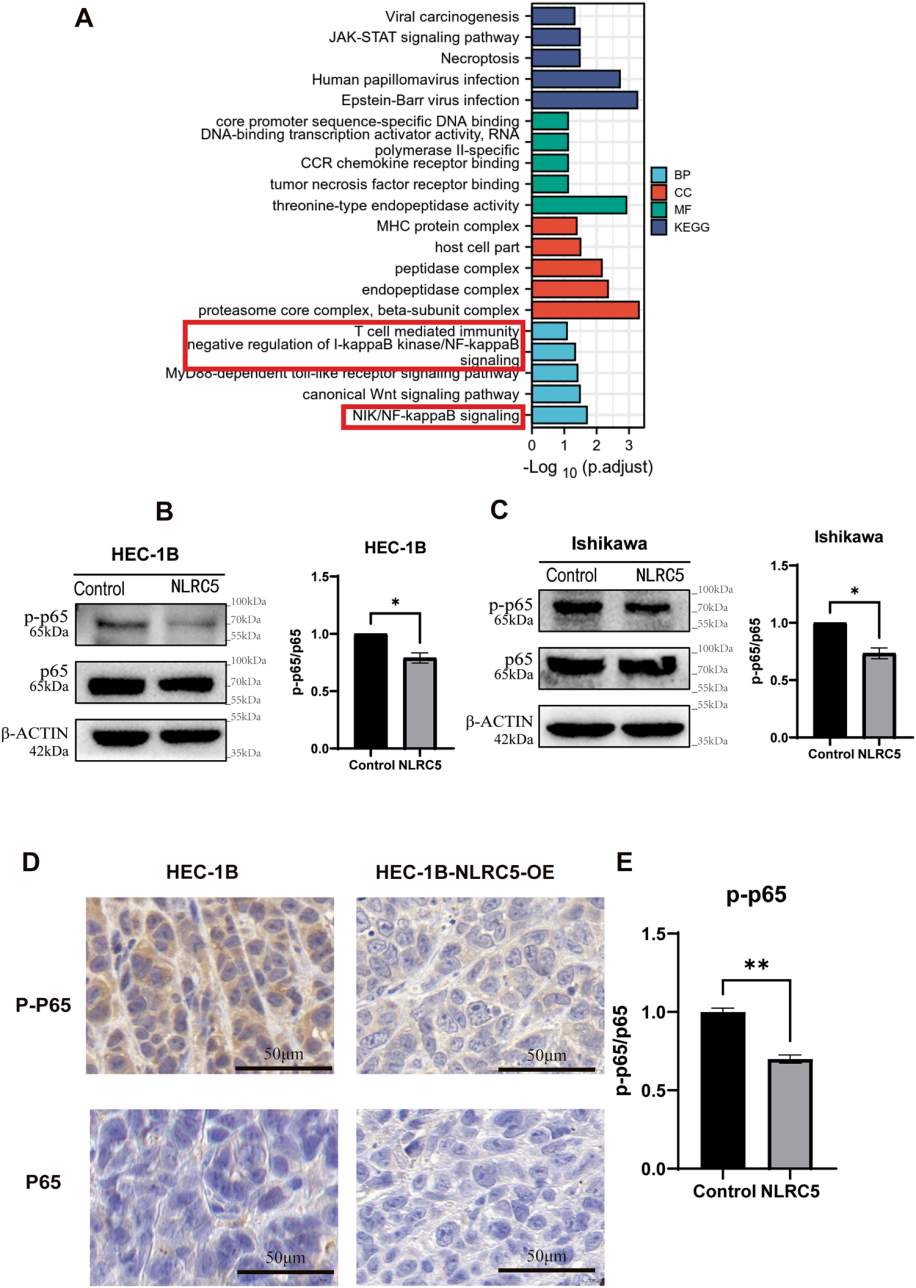
NLRC5 suppresses the NF-κB pathway in vivo and in vitro. (**A**) NLRC5-related pathways investigated by gene ontology (GO) terms and KEGG pathways enrichment scores. (**B**,**C**) The protein expression levels of p65 and p-p65 in HEC-1B and Ishikawa cell lines were examined by western blot. The protein expression levels were quantified using the ImageJ software and normalized using p65 protein levels (mean ± SD, n = 3). (**D**,**E**) Representative images of results of IHC detection of p65 and p-p65 protein expression of xenograft tumors. (Scale bar = 100 μm mean ± SD, n = 3). The corresponding brightfield images are included in the supplementary material. Data represent mean ± SD, *P < 0.05 versus control, **P < 0.01 versus control, ***P < 0.001 versus control.

We investigated NLRC5-related pathways using gene ontology (GO) terms and KEGG pathways enrichment scores, and found that NLRC5 may affect NF-κB, Wnt, JAK-STAT pathway and other signaling pathways (Fig. 4A). Several studies have confirmed that NLRC5 suppresses the NF-κB pathway in many diseases, but no study has so far established the relationship between NLRC5 and NF-κB in EC. In our study, protein expression was determined using western blotting to confirm that NLRC5 inhibited the activation of NF-κB in the EC cell lines HEC-1B and Ishikawa (Fig. 4B,C). In addition, the outcomes of the IHC assay of xenograft tumors in nude mice indicated that NLRC5 suppressed the NF-κB pathway in vivo (Fig. 4D,E).

### NLRC5 plays a pro-tumor role by suppressing the NF-κB pathway in EC

**Figure 5:**
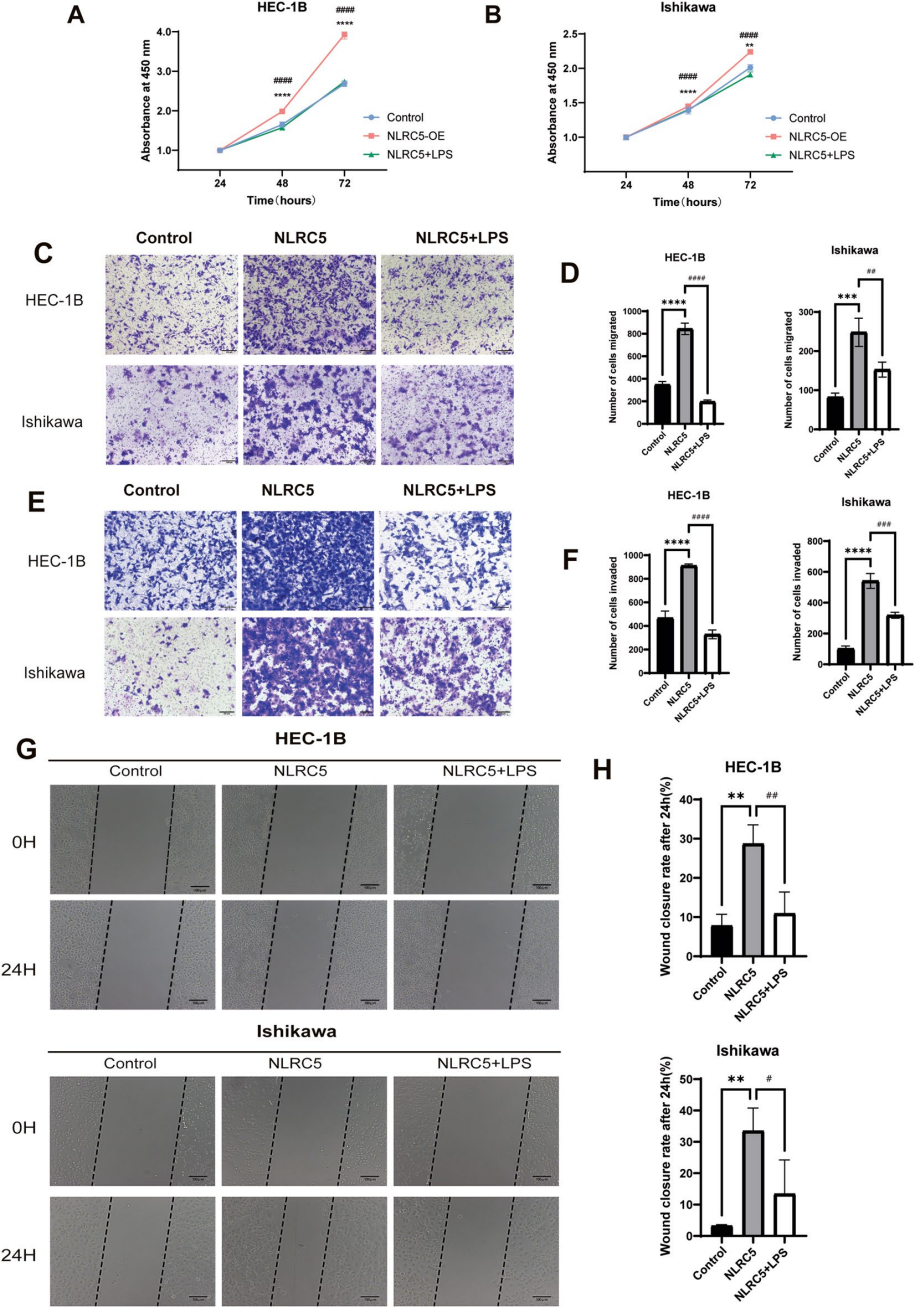
Activation of NF-κB pathway can inhibit the pro-tumor effect of NLRC5 on the proliferation, migration and invasion of EC cells. (**A**,**B**) The proliferation of HEC-1B cells and Ishikawa cells was measured via CCK-8 at 24,48 and 72 h. (**C**,**D**) Transwell assay detect the ability of cell migration (Scale bar = 100 μm, mean ± SD, n = 3). (**E**,**F**) Transwell assay detect the ability of cell invasion (Scale bar = 100 μm, mean ± SD, n = 3). (**G**) Wound healing detected the ability of cell migration(Scale bar = 100 μm, mean ± SD, n = 3). Data represent mean ± SD, ***P < 0.001 versus control, **P < 0.01 versus control, ****P < 0.001 versus control, ^##^*P* < 0.01 versus NLRC5, ^###^*P* < 0.001 versus NLRC5, ^####^*P* < 0.0001 versus NLRC5.

In the above experiments, NLRC5 was verified to induce dMMR in EC, thereby leading to increased proliferation, migration, and invasion of EC. To determine whether the protumor effect of NLRC5 on EC depended on the NF-κB pathway, LPS, an activator of NF-κB, was added to the EC cell lines overexpressing NLRC5. The results indicated that the pro-proliferative effect of NLRC5 was reversed (Fig. 5A,B). Subsequently, transwell (Fig. 5C–F) and scar-wound healing assays (Fig. 5G,H) were used to measure the cell migration and invasion abilities in each group. The results suggested that LPS inhibited the ability of NLRC5 in promoting the migration and invasion of tumor cells. These findings asserted that the protumor effect of NLRC5 on the proliferation, migration, and invasion of tumor cells depended on the suppression of the NF-κB pathway.

### NLRC5 induces dMMR in EC by suppressing the NF-κB pathway

**Figure 6:**
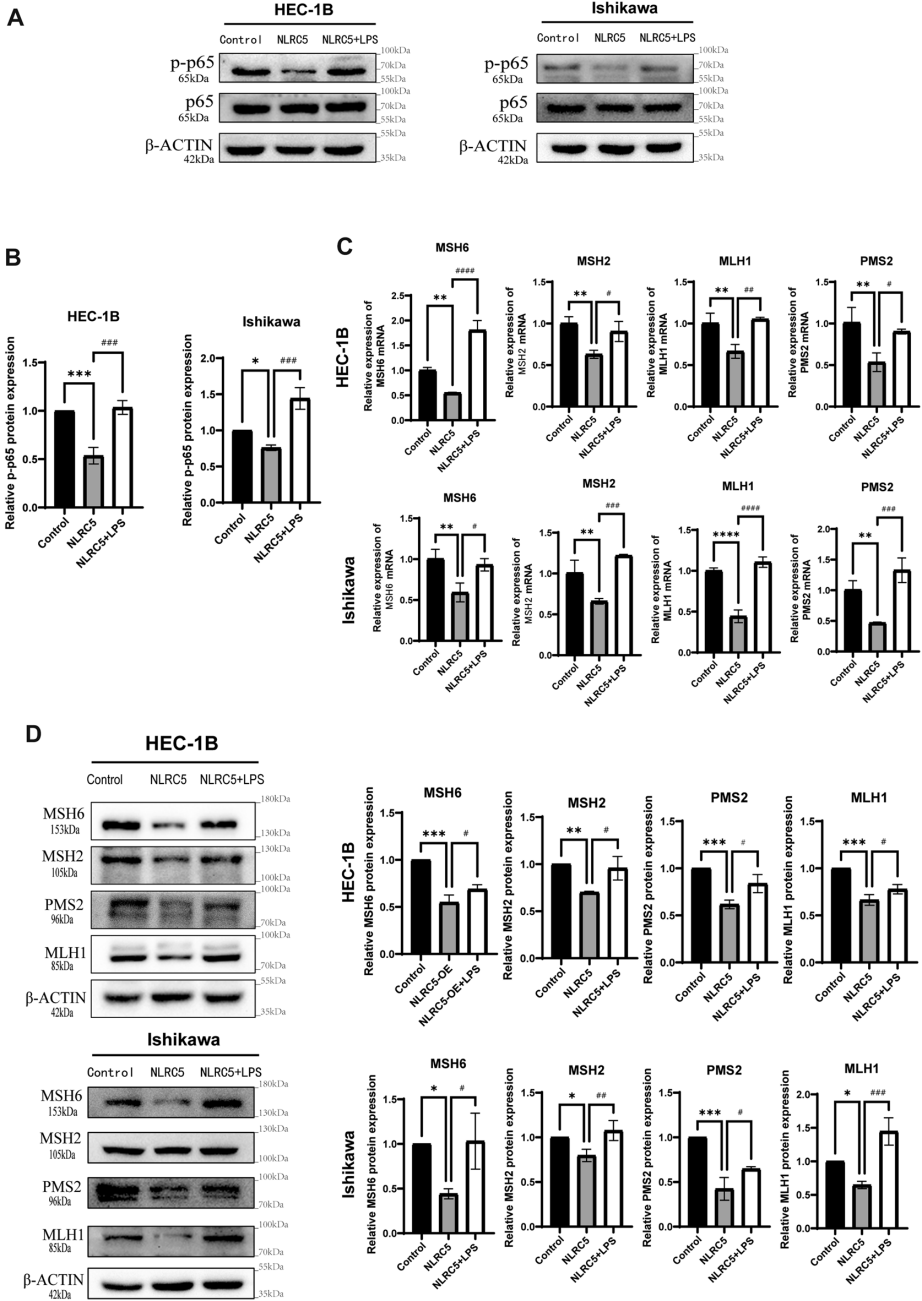
NLRC5 induces dMMR status by suppress NF-κB pathway in EC. (**A**,**B**) The protein expression levels of p65 and p-p65 in HEC-1B and Ishikawa cell lines were examined by western blot. The protein expression levels were quantified using the ImageJ software and normalized using p65 protein levels (mean ± SD, n = 3). (**C**) The mRNA expression levels of MMR genes in HEC-1B and Ishikawa cell lines were examined by qRT-PCR (mean ± SD, n = 3). (**D**) The protein expression levels of MMR genes in HEC-1B and Ishikawa cell lines were examined by western blot. The protein expression levels were quantified using the ImageJ software and normalized using β-actin protein levels (mean ± SD, n = 3). The corresponding brightfield images are included in the supplementary material. Data represent mean ± SD, *P < 0.05 versus control, **P < 0.01 versus control, ***P < 0.001 versus control, ****P < 0.0001 versus control. ^*#*^*P* < 0.05 versus NLRC5, ^##^*P* < 0.01 versus NLRC5, ^###^*P* < 0.001 versus NLRC5, ^####^*P* < 0.0001 versus NLRC5.

To further explore whether the inhibitory effect of NLRC5 on MMR genes was achieved via suppression of the NF-κB pathway, LPS was added to the EC cell lines overexpressing NLRC5. The results showed that NF-κB was activated after the addition of LPS (Fig. 6A,B). Furthermore, the suppressive effect of NLRC5 on the expression of MMR genes was reversed (Fig. 6C,D), which indicating that NLRC5 induced dMMR by suppressing the NF-κB pathway in EC.

## Discussion

The NOD-like receptor (NLR) family is a group of intracellular receptors in the autoimmune system. Members of the NLR family contain a central nucleotide-binding and oligomerization domain. Members of this family were originally thought to induce inflammatory responses by initiating the formation of inflammasomes^30^. NLRC5 is a recently discovered members of NLR family, it has been demonstrated that NLRC5 played significant roles in many pathophysiology^31^. For example, NLRC5 was found to exert a significant role in anti-tumor immunotherapy by activating immunosurveillance in immunomicroenvironment^16^. Moreover, NLRC5 is also closely related to inflammatory response regulation. The lack of NLRC5 has been proven to lead to the secretion of some inflammatory cytokines, such as tumor necrosis factor-alpha and interleukin-1b^32,33^. Additionally, several studies have found that NLRC5 plays a critical role in promoting the occurrence and development of tumors^19,34^. In gastric cancer, NLRC5 enhances the growth of malignant tumors by promoting the carcinogenic effect of YYI transcription factors^18^. In non-small cell lung cancer, NLRC5 promotes tumor progression by activating the PI3K/AKT pathway, thereby leading to carboplatin resistance^35^. Therefore, we believe that the exact role of NLRC5 was tissue type or microenvironment dependent. In our study, we found NLRC5 could promote the proliferation, migration, and invasion of EC cells. This conclusion was further confirmed in the mice xenograft model. Nevertheless, the inconsistent role of NLRC5 in different microenvironment of cancer, such as in EC, need to be investigated.

Histological classification of EC provides vital prognostic information to help determine appropriate surgery, and adjuvant therapy^36^. However, this classification is considered highly subjective with overlapping immunohistochemical and morphological features among EC subtypes in clinical settings. Based on TMB and somatic copy number variation, TCGA is classified EC into POLE ultra-mutated, dMMR, a copy-number low group with a low mutational burden, and a copy-number high serous-like group^2,3^.

Clinical trials have confirmed that MMR is a potential biomarker for predicting the response to tumor immunotherapy in EC^37,38^. One the one hand, dMMR tumors could produce excess new immunogenic antigens, which results in the increased permeability of CD8^+^ lymphocytes and the upregulation of genes encoding immune checkpoints, such as programmed cell death protein 1, PD-L1, and cytotoxic T-lymphocyte antigen 4^7,8^. One the other hand, dMMR also leads to the mutation and activation of several cancer-related genes, which enhances tumor proliferation, migration, and invasion abilities and promotes tumor progression^5^. Whereupon, we speculate that whether the inconsistent role of NLRC5 in different microenvironment of cancer was dependent on regulating dMMR. In this study, the overexpression of NLRC5 was shown to induce dMMR of EC, which was verified in vivo and in vitro. To determine whether the effect of NLRC5 on dMMR of EC was achieved via the inhibition NF-κB, the NF-κB activator LPS was added to the NLRC5-overexpressing cell lines. The results showed that the activation of NF-κB can reverse the effect of NLRC5 on the dMMR status of EC and inhibit the proliferation, migration, and invasion of cancer cells. This finding proves that NLRC5 can induce dMMR by suppressing the activation of NF-κB and promoting the development of EC. The MMR deficiency is often associated with promoter methylation and inflammation, especially the promoter methylation of MLH1^10,11,39^, But further research is needed to determine whether NF-κB affect MMR promoter methylation through inflammatory pathways, thereby affecting tumor dMMR status.

NLRC5 acts as a transactivator of MHC I, and tumors can achieve immune escape by targeting and inhibiting the expression of NLRC5^40^. Research has shown that NLRC5 is a biomarker for predicting the outcome of CTLA-4 blockade therapy^41^, NLRC5 overexpression is associated with CD8^+^ cytotoxic T cell activity^14,15^, promote the immunogenicity of tumors in human cancers^16^. However, in many studies, NLRC5 has been shown to greatly promote the occurrence and development of tumors in a nonimmune infiltration environment^18–21^. Similarly, a deficiency of MMR promotes tumor development on the one hand and causes high sensitivity to immune agents on the other hand. This could be attributed to the increase in the tumor mutation load, which in turn leads to the production of related new antigens^8,9^., we guess that NLRC5 may activate immune checkpoint and suppress immune escape by promoting dMMR of EC in the immune microenvironment, but further research is needed to prove this hypothesis.

## Conclusion

The findings prove that NLRC5 induces dMMR in EC and promotes tumor proliferative capacity in vitro and in vivo. Investigation of the regulatory mechanism of the protumor effect of NLRC5 on EC revealed that NLRC5 suppresses the NF-κB pathway in vitro and in vivo. When NF-κB was activated, the protumor effect of NLRC5 on EC was reversed, which is akin to the dMMR status induced by NLRC5. These findings suggest that NLRC5 promotes EC progression by regulating the NF-κB pathway-mediated dMMR.

### Supplementary Information

Supplementary Information.

## Data Availability

The datasets used and/or analysed during the current study are available from the corresponding author on reasonable request.
