# Supplementary material for: NLRC5 promotes endometrial carcinoma progression by regulating NF-κB pathway-mediated mismatch repair gene deficiency
Source: Sci Rep. 2024 May 30;14:12447. doi: 10.1038/s41598-024-63457-2 (PMC11143240; doi:10.1038/s41598-024-63457-2)

Manuscript ID: 747de29b-d496-4bad-be20-fecfc9337f2f

Title: NLRC5 promotes endometrial carcinoma progression by regulating NF-κB pathway-mediated mismatch repair gene deficiency

Raw data of Western blot in Figure 1A

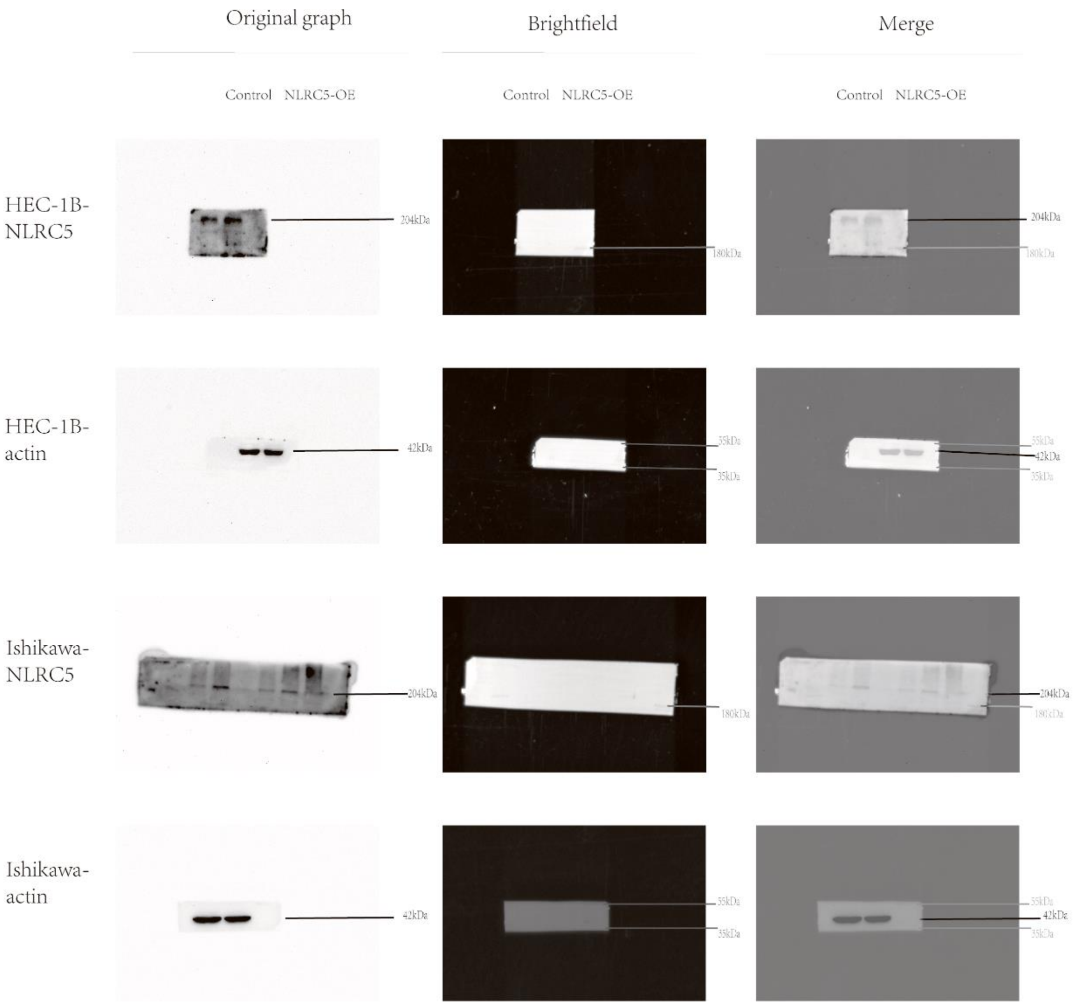

Raw data of Western blot in Figure 2C

HEC-1B

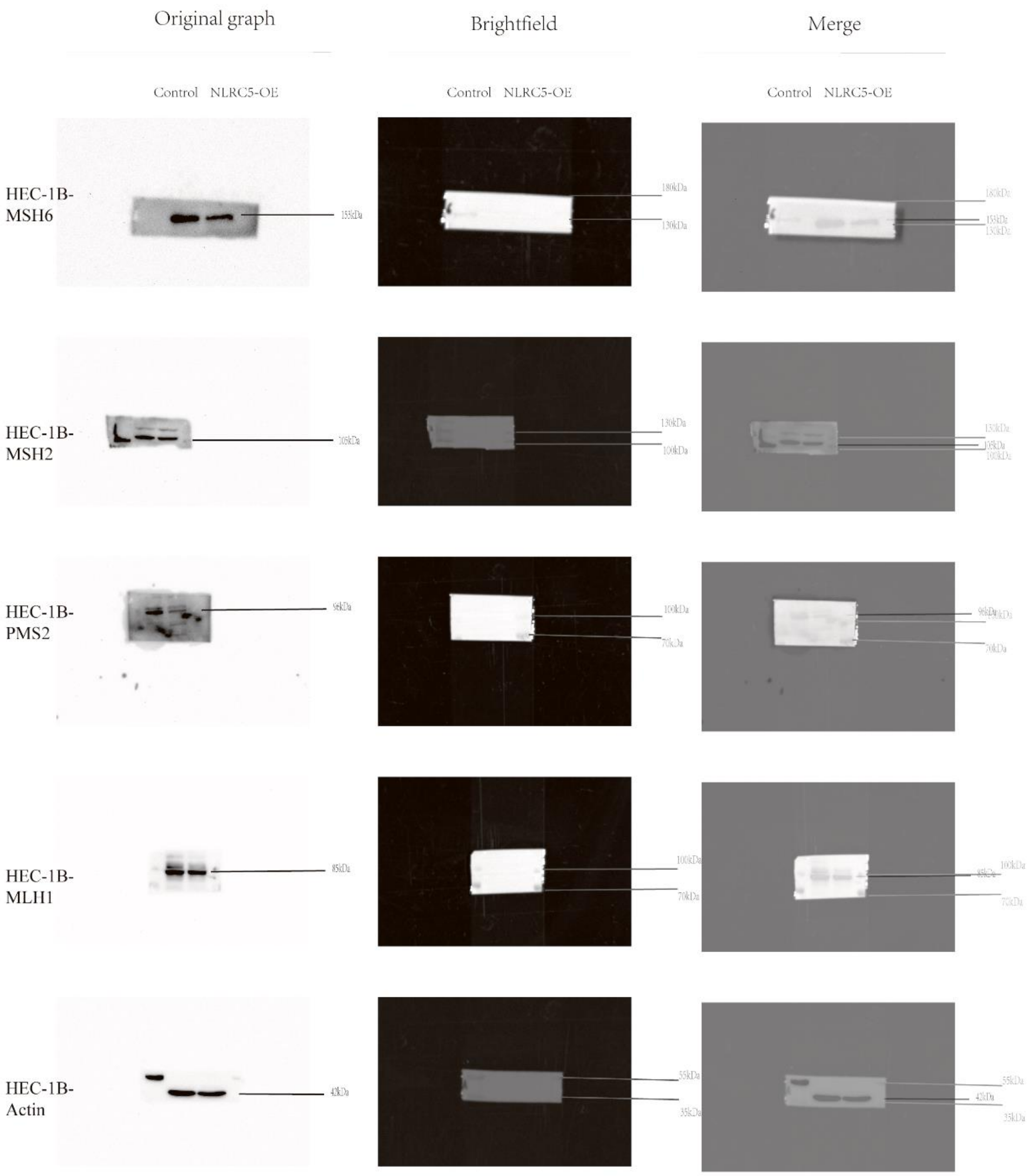

Raw data of Western blot in Figure 2D

Ishikawa

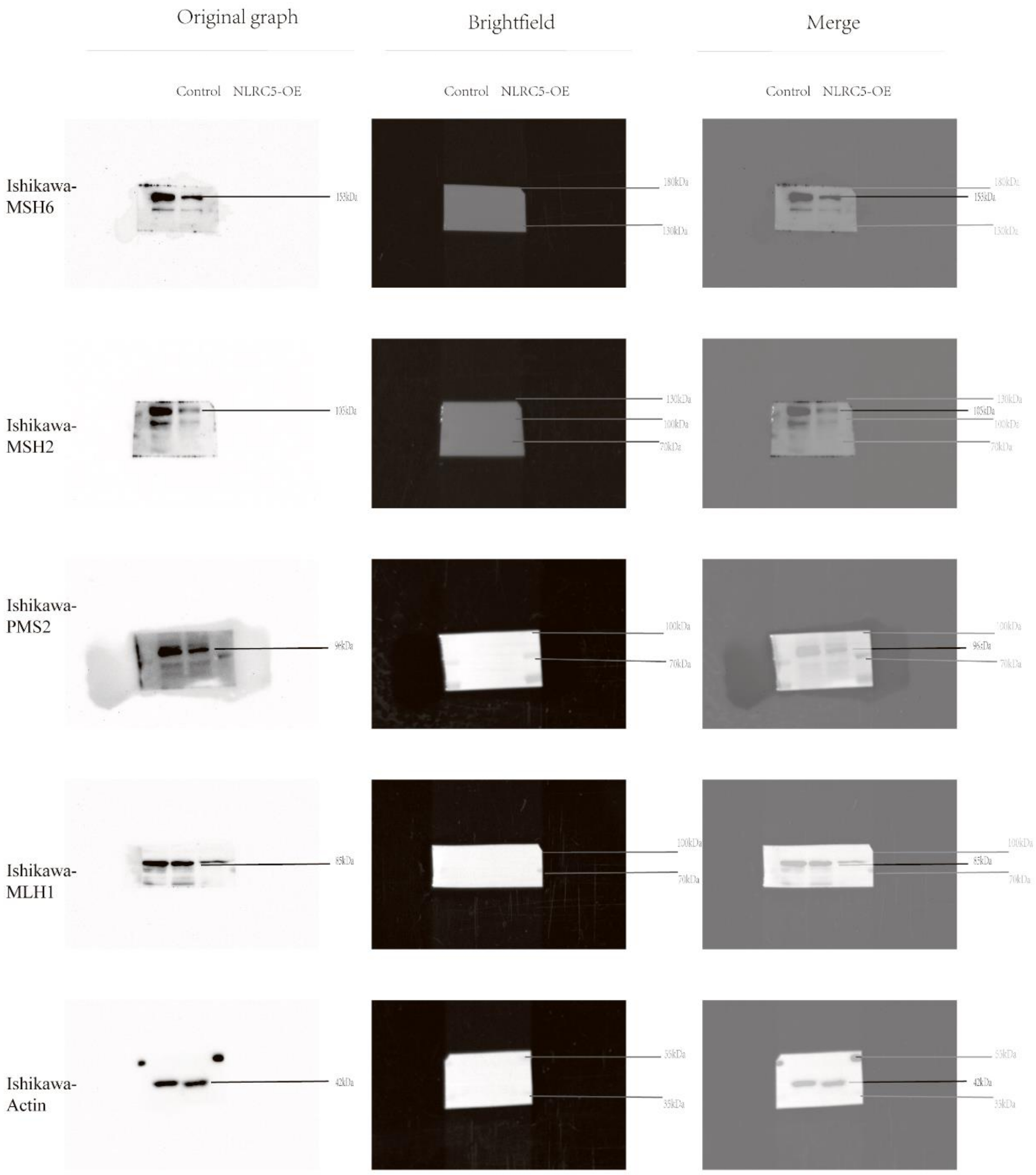

Raw data of Western blot in Figure 4B

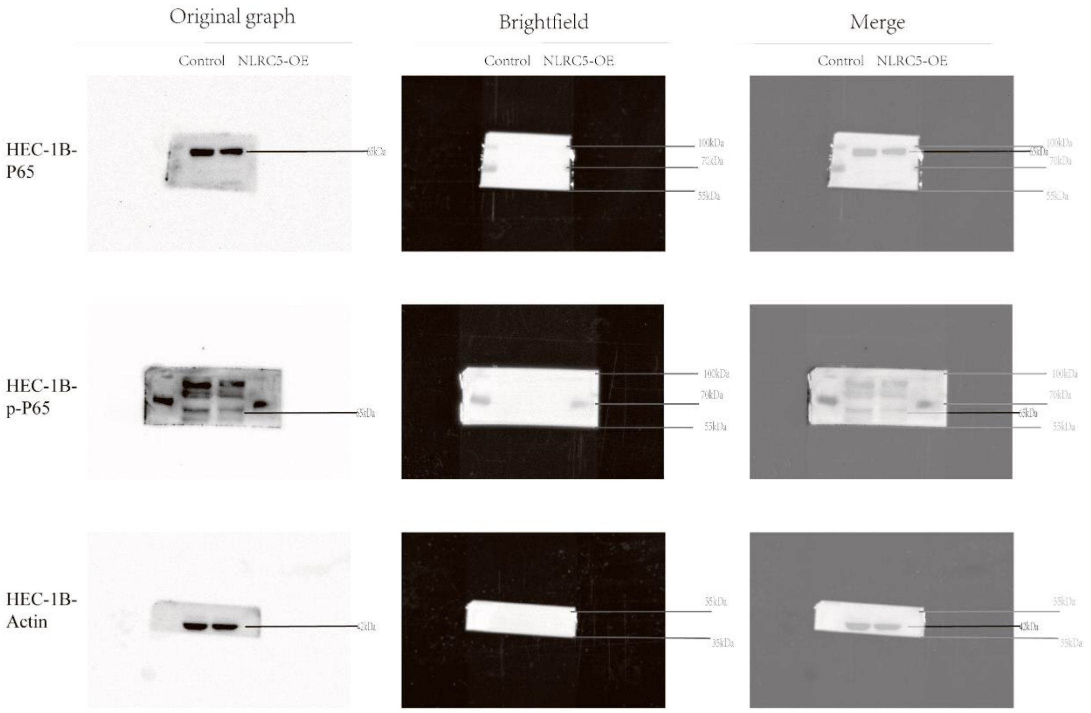

Raw data of Western blot in Figure 4C

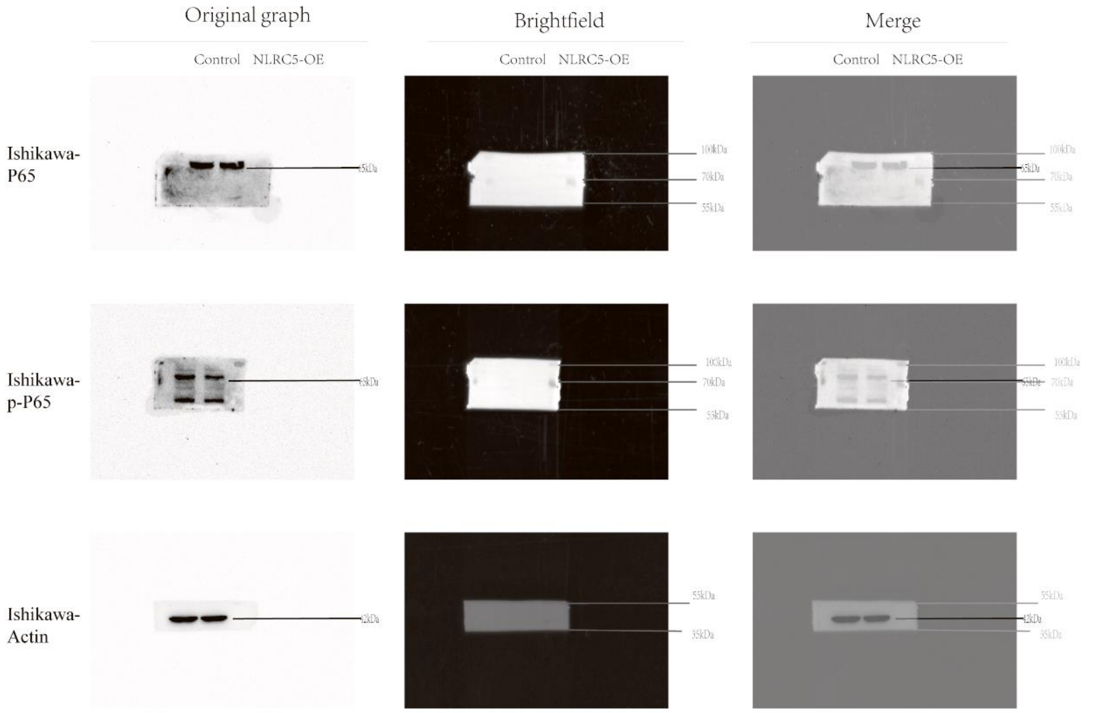

Raw data of Western blot in Figure 6A

HEC-1B

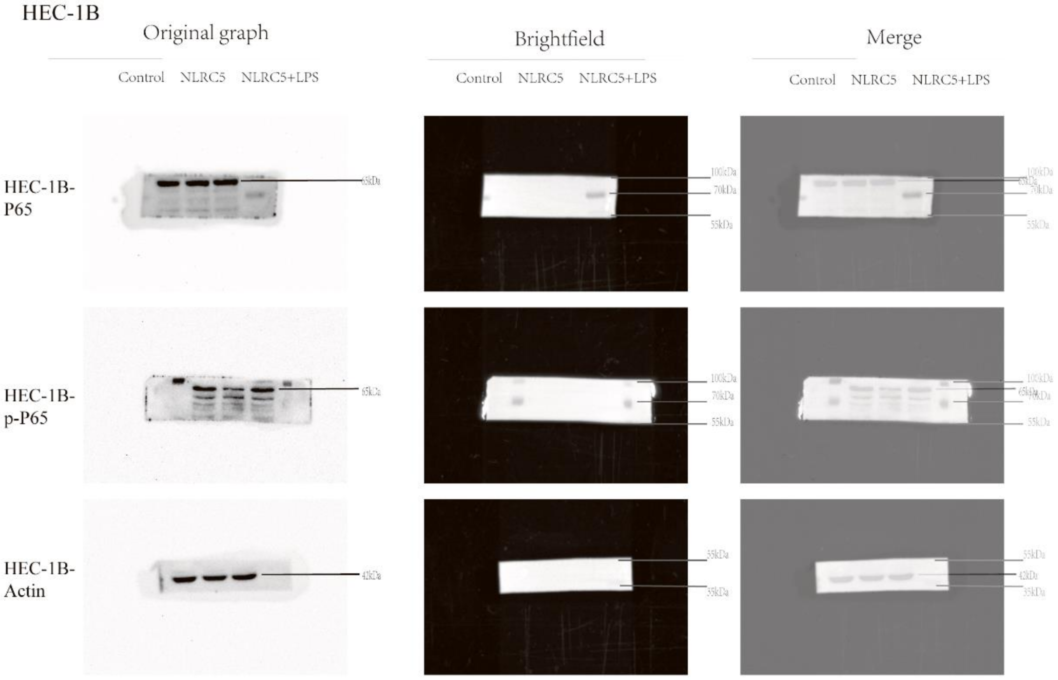

Raw data of Western blot in Figure 6A

Ishikawa

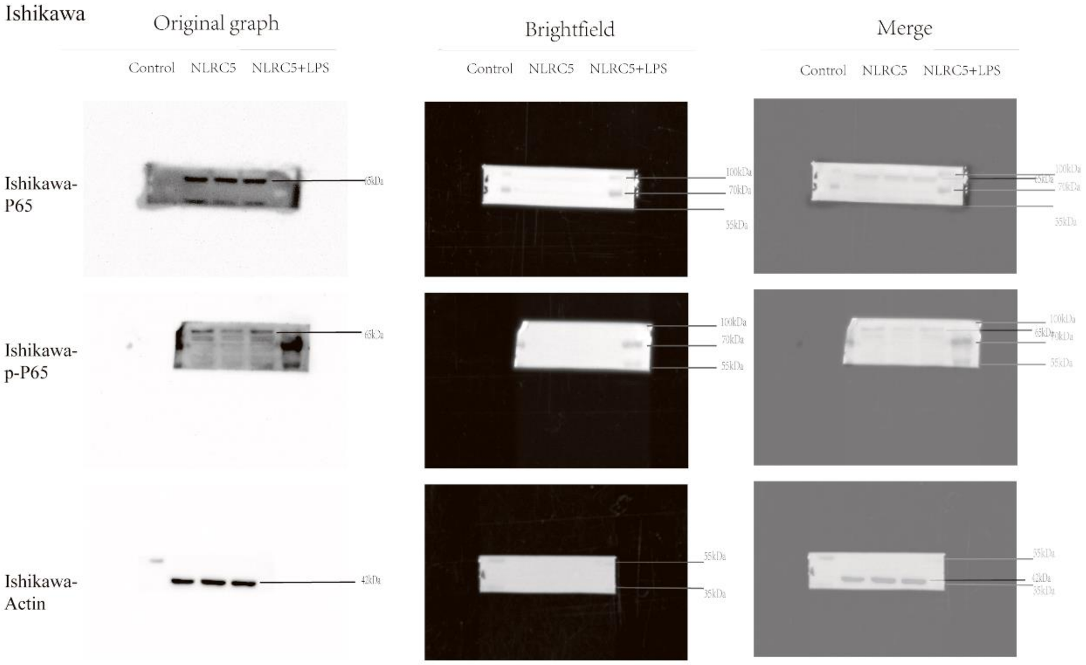

Raw data of Western blot in Figure 6D

HEC-1B

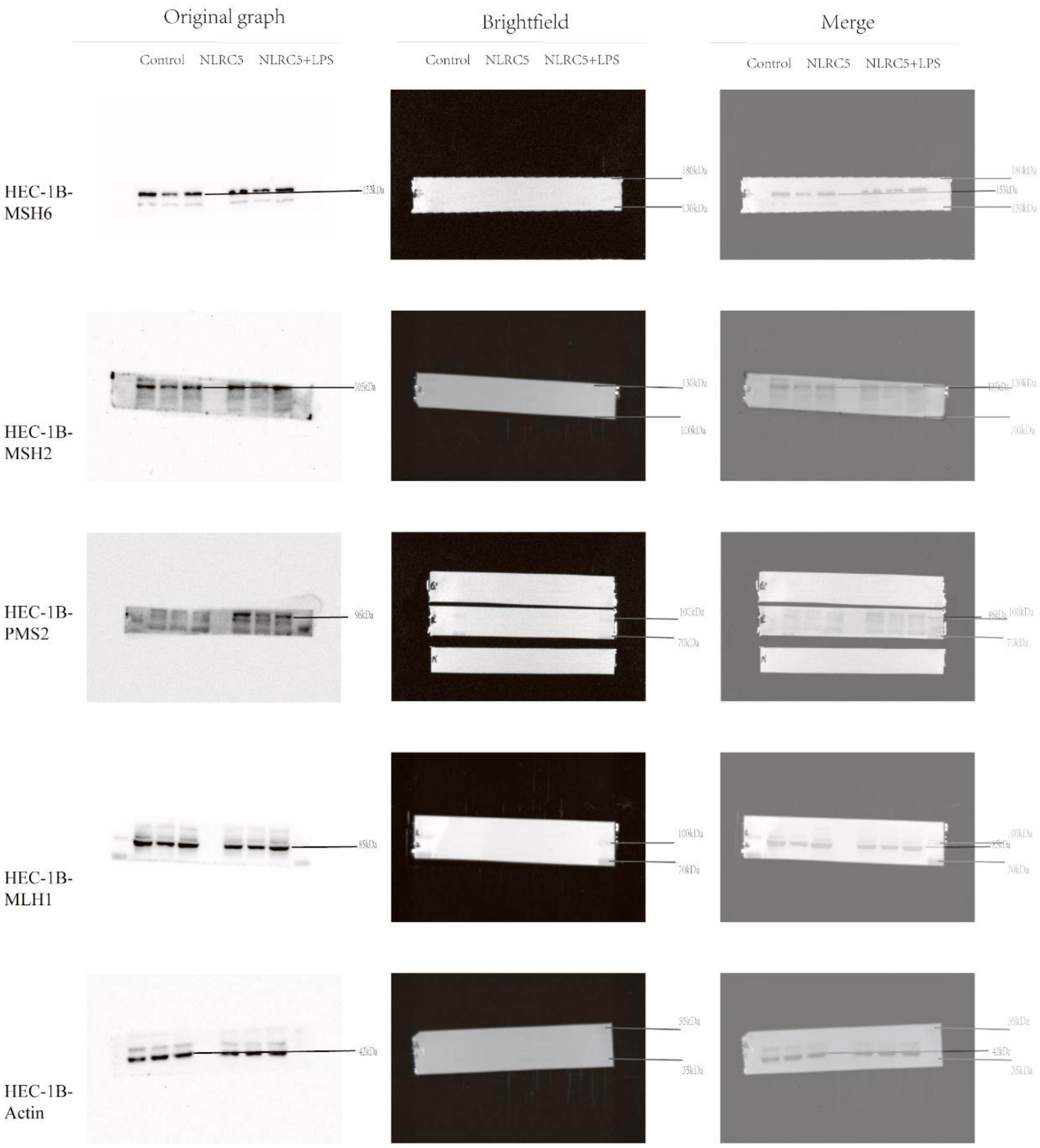

Raw data of Western blot in Figure 6D

Ishikawa

Ishikawa-MSH6

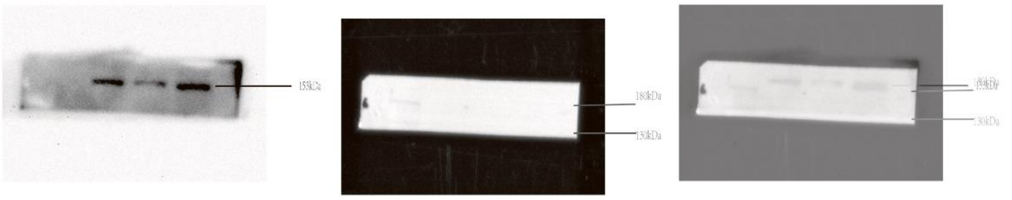

Ishikawa-MSH2

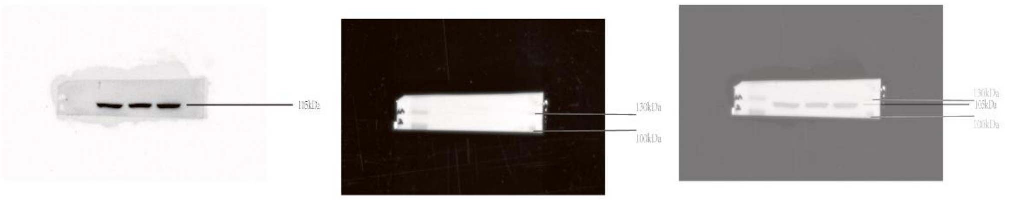

Ishikawa-PMS2

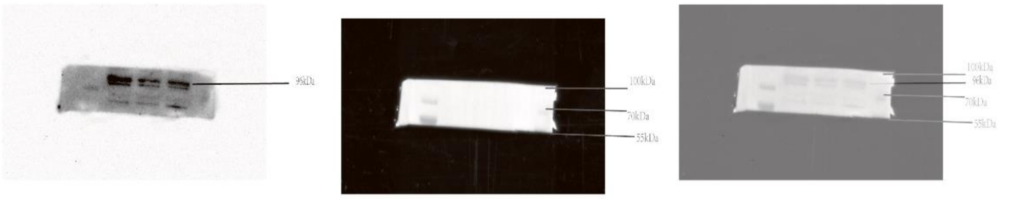

Ishikawa-MLH1

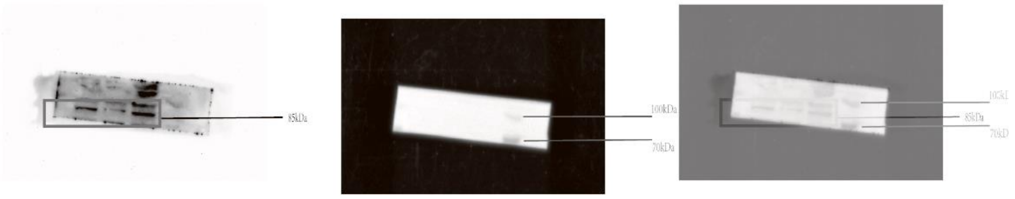

Ishikawa-Actin

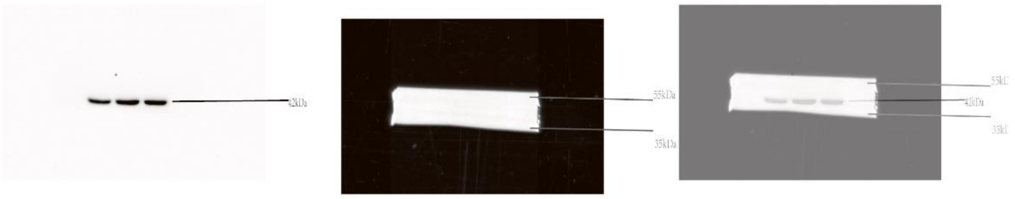

Supplement: Supplementary file 1 — Supplementary Information. [file 41598_2024_63457_MOESM1_ESM.pdf]
